# A novel consensus bacterial 6-phytase variant supplemented to an all-vegetable broiler diet totally replaced added trace minerals including zinc, iron, copper and manganese in two experiments

**DOI:** 10.1016/j.psj.2024.104610

**Published:** 2024-11-27

**Authors:** Yueming Dersjant-Li, Cees Kwakernaak, Abiodun Bello, Leon Marchal

**Affiliations:** a Danisco Animal Nutrition & Health (IFF), Willem Einthovenstraat 4, 2342 BH Oegstgeest, the Netherlands; b Schothorst Feed Research, PO Box 533, 8200 AM Lelystad, The Netherlands; c Danisco Animal Nutrition & Health, IFF, Wilmington, 19803, DE, USA

**Keywords:** Broilers, Phytase, Trace minerals, Bio-availability

## Abstract

Two experiments tested the effect of a bacterial 6-phytase (**PhyG**) supplemented to a vegetable broiler diet without or with added trace minerals (**TM**), on growth performance and TM utilization. Each tested 12 treatments in a 2 × 6 factorial arrangement with 3,360 Ross 308 males (35 birds/pen, 8 pens/treatment) in a randomized complete block design. Phytase levels comprised no PhyG or PhyG at 2,000, 1,500 and 1,000 FTU/kg during 0 to 10, 10 to 20 and (Experiment 2) 20 to 35 d of age, respectively; TM diets comprised: no added TM (diet 1); 10, 15, 3 and 10, or 20, 30, 6 and 20 mg/kg of Zn, Fe, Cu and Mn as sulphate (diets 2 and 3); 15, 3 and 10 mg/kg of Zn, Cu and Mn as oxide and 15 mg/kg Fe as sulphate, or 30, 6 and 20 mg/kg of Zn, Cu and Mn as oxide and 30 mg/kg Fe as sulphate (diets 4 and 5), and; 10, 3, 15 and 10 mg/kg of organic Zn, Cu, Fe and Mn (diet 6). Apart from no added TM, basal diets were nutritionally adequate; phytase-supplemented diets were reduced in Ca and retainable P. All diets were pelleted and fed ad libitum. In both experiments during all phases there were interactions (*P* < 0.05) between phytase and TM on BW, BW gain (**BWG**), feed intake (**FI**), FCR (experiment 2 only) and tibia Zn. Without phytase, TM improved (*P* < 0.05) these measures. Phytase without TM increased (*P* < 0.05) d 35 BW, overall BWG and FI, equivalent to or beyond the level achieved by TM without phytase. Tibia Zn at d 10 and 20 in Experiment 2 was increased by phytase beyond the level achieved by TM without phytase (*P* < 0.05). Phytase also increased (*P* < 0.05) liver Zn, Fe, and Mn at d 10 and 20 and plasma Zn at d 20 d. There was no consistent effect of TM source or dose on bird responses. In conclusion, PhyG phytase replaced the effect of added TM on performance during 0 to 35 d of age and could support a reduction in commercial dose levels of added TM in all-vegetable broiler diets.

## Introduction

Trace minerals (**TM**) are essential for the healthy development of bones, enzyme structure and function, and for catalyzing biological reactions (Suttle, 2010). Their availability in poultry feedstuffs tends to be variable and is considered insufficient to meet bird requirements. Hence, commercial broiler diets are typically supplemented with Zn, Fe, Cu, Mn and Se, via a TM premix. The amounts added are variable, reflecting the wide variation in recommendations by breeders and affiliations such as the NRC (1994), and tend to include a considerable safety margin. Reducing TM supplementation would reduce the likelihood of TM excess occurring in the gastrointestinal tract (**GIT**) which can negatively affect the digestion of dietary constituents (e.g. phytate). It may also reduce TM excretion which could improve the environmental impact of broiler production. One approach to reducing TM supplementation is to increase the availability and utilization of the TM contained within dietary ingredients.

Phytate (*myo*-inositol hexakisphosphate, **IP_6_**) is the major storage form of P in plant ingredients (Selle and Ravindran, 2007). It has a strong affinity to bind with mineral cations including Ca^2+^, Zn^2+^, Fe^2+^, Mn^2+^, Mg^2+^, Cu^2+^ and Co^2+^ in the pH environment (pH 4-6) of the small intestine (Selle and Ravindran, 2007; Selle et al., 2009), forming insoluble complexes that are not readily digested. Undigested phytate can therefore significantly reduce mineral availability for absorption. This has been shown extensively for Ca as the most abundant mineral in the diet, where one phytate molecule (as IP_6_) can bind up to five Ca atoms in the broiler small intestine (Selle et al., 2009) but also for Zn (Augspurger et al., 2004), and Fe (Sandberg et al., 1999; Yu et al., 2012).

Exogenous microbial phytase is already widely added to broiler diets to improve P availability in the diet. This is achieved via the stepwise hydrolysis of phytate which releases inorganic phosphate. Phytase efficacy in improving P digestibility and utilization in poultry is well proven (Selle and Ravindran, 2007; Humer et al., 2015). Its capacity to simultaneously improve the digestibility of energy, sodium, digestible amino acids, and proteins has also been extensively demonstrated (Sebastian et al., 1997; Ravindran et al., 2006; Liu et al., 2015; Truong et al., 2015; Dersjant-Li et al., 2022a). Phytase effects on TM availability and utilization have been less studied but a beneficial effect is predicted by the known capacity of TM to bind to phytate. In vitro research has shown that phytase can ‘release’ Cu, Zn, Fe and Mn from individual feed ingredients (Yu et al., 2018) and a small number of in vivo studies have shown increased Zn availability and utilization in broiler diets supplemented with phytase (Jondreville et al., 2007; Schlegel et al., 2013). Further studies using current generation phytases are needed to determine their efficacy to improve TM availability wider than Zn, and to establish whether phytase supplementation could totally replace the beneficial effects of supplemental TM at tissue and bird level. In such studies it is important to study effects on multiple TM simultaneously, because individual TM may compete for uptake pathways in the GIT (Cousins, 1985; Spencer et al., 1994) so that changes in the availability of one TM could affect the uptake of another. When evaluating the effect of phytase on TM availability and utilization, it may also be relevant to consider the Ca level of the diet because an excess of Ca can act antagonistically with other macrominerals and TM, impeding their absorption and utilization (Smith and Kabaija, 1985; Shafey, 1993; Faria et al., 2020). In addition, because Ca has high affinity to bind to phytate, high dietary Ca can reduce phytase efficacy (Tamim et al., 2004; Li et al., 2017).

The hypothesis of this study was that dietary supplementation with a novel consensus bacterial 6-phytase variant (**PhyG**) would improve the availability and utilization of TM in a commercial all-vegetable broiler diet to a level comparable to a diet containing added TM without phytase. Experiment 1 was performed using a basal diet of high Ca content, whereas Experiment 2 employed a diet of Ca content meeting breeder recommendations. Both used identical study designs. The impact of phytase supplementation compared to TM supplementation on growth performance and tissue TM utilization was investigated, as well as the impact of TM source and supplementation level (low or high).

## Materials and methods

Both experiments were carried out at Schothorst Feed Research (Lelystad, the Netherlands). The research was conducted in accordance with European Directive 2010/63/EU and the regulations in force in the Netherlands for the care and use of animals in research and the experimental protocols and procedures were evaluated and approved by the Ethical Committee on Animal Experiments (Ethische Toetsing Dierproeven).

### Birds and housing

In both studies, Ross 308 male broilers were obtained on day-of-hatch from a commercial hatchery where they had been vaccinated against infectious bronchitis. Birds were assigned to pens based on body weight (**BW**) so that each pen contained birds of approximately equal average bird weight. All birds were vaccinated against Newcastle disease at 7 d of age. Pens were in a broiler house that contained wood shavings as bedding. Temperature was maintained initially at 34 °C and gradually reduced to 22 °C by d 24, 21 °C by d 29, 20 °C by d 32 and 19 °C by d 35 d. The lighting regime was LD 23:1h on d 0 and thereafter 3L:1D:12L:4D:3L:1D to stimulate mobility and thus prevent potential leg problems as part of the ethics approval. Diets and water were provided ad libitum during all phases.

### Trial design and dietary treatments

Both experiments were carried out as 2 × 6 factorial arrangements, each with two phytase levels and six TM diets in a randomized complete block design. The location of pens in the animal house was the blocking factor. In each experiment, there were 12 dietary treatments, eight replicate floor-pens (each pen 2 m^2^), 35 birds per pen and 3,360 birds in total (6,720 birds across both experiments). The treatments are summarized in Table 1 and described below.

**Table 1:** Treatment details (Experiment 1 and 2).

Treatment	Phytase (±)^1^	Added trace minerals (mg/kg feed during all phases)						
		Source	Level^2^	Zn	Cu	Fe^3^	Mn	Se^4^
1 (= CON1)	-	-	-	-	-	-	-	-
2	-	Sulphate-based	Low	10	3	15	10	0.15
3	-	Sulphate-based	High	20	6	30	20	0.15
4	-	Oxide-based	Low	15	3	15	10	0.15
5	-	Oxide-based	High	30	6	30	20	0.15
6	-	Organic-based	Low	10	3	15	10	0.15
7 (= CON2)	+	-	-	-	-	-	-	-
8	+	Sulphate-based	Low	10	3	15	10	0.15
9	+	Sulphate-based	High	20	6	30	20	0.15
10	+	Oxide-based	Low	15	3	15	10	0.15
11	+	Oxide-based	High	30	6	30	20	0.15
12	+	Organic-based	Low	10	3	15	10	0.15

1 Included at 2,000, 1,500 and 1,000 FTU/kg in starter, grower and finisher phases, respectively.

2 ‘High’: representing a marginal level of TM expected to achieve normal growth performance in broilers based on the available literature; ’Low’: reduced by 50 % vs. ‘high’.

3 FeSO_4_ was used in place of FeO in treatments 4,5,10 and 11, because FeO is not currently permitted for use as a trace mineral supplement in animal feed (EFSA, 2016).

4 Se was added in the form of Na_2_SeO_3_ in all treatments and was added at one dose level only because the analytical uncertainty associated with such a low dose level did not enable to distinct dose levels to be reliably applied.

In Experiment 1, diets were formulated in two phases: 0 to 10 d of age (starter) and 10 to 20 d of age (grower), whereas in Exp. 2, diets were formulated in three phases: 0 to 10 d of age (starter), 10 to 20 d of age (grower) and 20 to 35 d of age (finisher). All diets were based on corn and soybean meal with added rice bran, corn gluten meal, and (in finisher phase in Exp. 2 only) rapeseed meal and sunflower meal. Titanium dioxide (3.5 g/kg) was added during grower phase as an indigestible marker for potential measurements of nutrient digestibility that were not ultimately undertaken. The two phytase levels comprised no PhyG or PhyG supplemented to the basal diet at 2,000, 1,500 and 1,000 FTU/kg during 0 to 10, 10 to 20 and (only in Experiment 2) 20 to 35 d of age, respectively. These levels are within the current range of phytase supplementation levels used commercially. The phytase inclusion was based on the analyzed activity of the phytase in the product with 10 % extra to account for mixing errors. The six TM diets comprised: no added TM (diet 1); Zn, Fe, Cu and Mn as sulphate added at ‘low’ level (10, 15, 3 and 10 mg/kg, respectively; diet 2) or ‘high’ level (20, 30, 6 and 20 mg/kg, respectively; diet 3); Zn, Cu and Mn as oxide and Fe as sulphate, added at ‘low’ level (15, 3, 10 and 15 mg/kg, respectively; diet 4) or ‘high’ level (30, 6, 20 and 30 mg/kg, respectively; diet 5), and; Zn, Cu, Fe and Mn in organic form added at ‘low’ level (10, 3, 15 and 10 mg/kg, respectively; diet 6). The organic forms of Zn, Cu, Fe and Mn were chelates of amino acids hydrates. The levels of these individual TM in the ‘high’ and ‘low’ treatments were selected based on the TM range within which a dose-response effect (on TM utilization in tissues or growth performance) has been observed in the existing literature and where the amount of Zn supplemented in the ‘high’ dose level treatments was expected to be a marginal level to achieve normal growth performance in birds (Mohanna and Nys, 1999; Jondreville et al., 2007). It is acknowledged that commercial levels of TM supplementation can be variable and are often well in excess of NRC recommendations. Selenium, as sodium selenite (Na_2_SeO_3_) was supplemented at the same level (0.15 mg/kg) across all of diets 2–6, this representing the requirement level recommended by the NRC (NRC, 1994).

In both experiments, the basal diet of treatments 1–6 that contained no added phytase was named CON1 and was formulated to meet the recommendations for broilers (CVB, 2021) except without added TM and with a Ca content that was ‘high’ (above breeder recommendations) in Experiment 1 (Ca content 1.14 and 1.06 % during 0 to 10 and 10 to 20 d of age, respectively), and ‘normal’ (approximating breeder recommendations and current industry standards) in Experiment 2 (0.92, 0.84 and 0.77 % during 0 to 10, 10 to 20 and 20 to 35 d of age, respectively). The basal diet of treatments 7 to 12 that contained added PhyG was named CON2 and was reduced in Ca and retainable P vs. CON1 to account for the expected contribution of the supplemental phytase [by 0.23, 0.22 and 0.21 and 0.19, 0.18 and 0.16 % points, during 0 to 10, 10 to 20 and (Experiment 2 only) 20 to 35 d of age, respectively]. The reductions in Ca and P in CON2 were achieved by removal of some of the limestone and monocalcium phosphate and replacement with silicate as a filler. All diets were pelleted and fed ad libitum. The full ingredient composition and calculated nutrient content of the CON1 and CON2 diets in both studies is given in Table 2.

**Table 2:** Ingredient, calculated and analyzed nutrient composition of the basal diets in Experiment 1 and 2.

	Experiment 1				Experiment 2					
	Starter (0 to 10 d of age)		Grower (10 to 20 d of age)		Starter (0 to 10 d of age)		Grower (10 to 20 d of age)		Finisher (20 to 35 d of age)	
	CON1^1^	CON2^2^	CON1^1^	CON2^2^	CON1^1^	CON2^2^	CON1^1^	CON2^2^	CON1^1^	CON2^2^
Ingredients, % as fed										
Corn	57.0	57.0	56.7	56.7	58.2	58.2	57.7	57.7	63.2	63.2
Soybean meal	28.3	28.3	29.1	29.1	28.6	28.7	29.4	29.4	18.28	18.28
Rice bran	2.86	2.86	2.94	2.94	2.82	2.83	3.23	3.23	-	-
Corn gluten meal	3.42	3.42	2.00	2.00	3.00	3.00	1.00	1.00	-	-
Sunflower seed meal	-	-	-	-	-	-	-	-	6.00	6.00
Rapeseed meal	-	-	-	-	-	-	-	-	3.60	3.60
Soybean oil	1.00	1.00	3.28	3.28	2.01	2.01	3.44	3.44	2.75	2.75
Limestone	2.15	1.96	2.03	1.84	1.23	1.10	1.11	0.97	1.04	0.90
Monocalcium phosphate	1.61	0.61	1.40	0.44	1.61	0.60	1.41	0.44	1.11	0.22
Silicate	0.00	1.19	0.00	1.14	-	1.15	-	1.10	-	1.03
Lard	1.19	1.19	0.28	0.28	-	-	0.19	0.19	2.10	2.10
Salt	0.33	0.33	0.34	0.34	0.20	0.20	0.07	0.07	0.26	0.26
L-lysine HCl	0.42	0.42	0.26	0.26	0.42	0.42	0.26	0.26	0.31	0.31
DL-methionine	0.31	0.31	0.26	0.26	0.31	0.31	0.27	0.27	0.17	0.17
L-threonine	0.11	0.11	0.09	0.09	0.11	0.11	0.09	0.09	0.06	0.06
L-tryptophan	0.03	0.03	0.01	0.01	0.03	0.03	0.01	0.01	0.01	0.01
L-arginine	0.13	0.13	-	-	0.12	0.12	-	-	-	-
L-valine	0.07	0.07	0.00	0.00	0.07	0.07	0.01	0.01	0.01	0.01
Titanium dioxide^3^	-	-	0.35	0.35	-	-	0.35	0.35	-	-
Sodium bicarbonate	-	-	-	-	0.20	0.20	0.39	0.39	0.10	0.10
Vitamin premix^4^	0.50	0.50	0.50	0.50	0.50	0.50	0.50	0.50	0.50	0.50
Trace mineral premix (corn only in controls)	0.50	0.50	0.50	0.50	0.50	0.50	0.50	0.50	0.50	0.50
Sacox	0.01	0.01	0.01	0.01	0.01	0.01	0.01	0.01	-	-
PhyG, FTU/kg^5^	-	2,000	-	1,500	-	2,000	-	1,500	-	1,000
Total	100	100	100	100	100	100	100	100	100	100
Chemical composition, % as is (unless otherwise stated)										
ME, kcal/kg	2,900	2,900	2,975	2,975	2,900	2,900	2,975	2,975	3,075	3,075
Moisture	11.7	11.7	11.6	11.6	11.7	11.7	11.7	11.7	11.6	11.6
Ash	6.2	6.2	6.2	6.2	6.05	6.05	5.99	5.99	5.33	5.33
Crude protein	21.1	21.1	20.1	20.1	21.1	21.1	19.8	19.8	17.8	17.8
Crude fat	5.5	5.5	6.8	6.8	5.30	5.30	6.83	6.83	7.96	7.96
Crude fiber	2.4	2.4	2.4	2.4	2.40	2.40	2.41	2.41	2.92	2.92
Calcium	1.14	0.92	1.06	0.85	0.92	0.69	0.84	0.62	0.77	0.56
Phosphorus (P)	0.72	0.50	0.68	0.47	0.72	0.50	0.67	0.46	0.61	0.42
Retainable phosphorus	0.43	0.24	0.39	0.21	0.43	0.24	0.39	0.21	0.33	0.17
Phytate-P	0.25	0.25	0.25	0.25	0.25	0.25	0.25	0.25	0.25	0.25
SID Lys	12.1	12.1	10.9	10.9	12.1	12.1	10.9	10.9	9.6	9.6
SID Met	6.0	6.0	5.4	5.4	6.0	6.0	5.4	5.4	4.4	4.4
SID Met+Cys	8.6	8.6	7.9	7.9	8.6	8.6	7.9	7.9	6.8	6.8
SID Thr	7.4	7.4	7.0	7.0	7.4	7.4	7.0	7.0	6.1	6.1
SID Trp	2.2	2.2	2.0	2.0	2.2	2.2	2.0	2.0	1.7	1.7
Zinc, mg/kg	28	27	27	27	28	27	28	27	30	29
Iron, mg/kg	147	103	137	94	150	107	150	109	148	110
Copper, mg/kg	5.4	5.4	5.4	5.3	5.4	5.4	5.0	5.0	5.8	5.8
Manganese, mg/kg	28	23	27	21.	28	23	27	22	24	19
Selenium, mg/kg	0.1	0.1	0.1	0.1	0.1	0.1	0.1	0.1	0.1	0.1
Analyzed nutrients, %										
Calcium	1.19	0.97	1.09	0.94	0.85	0.69	0.92	0.70	0.76	0.54
Phosphorus	0.73	0.50	0.69	0.47	0.83	0.54	0.70	0.48	0.66	0.42
Phytate -P	0.26	0.26	0.25	0.24	0.24	0.25	0.25	0.26	0.27	0.27

SID, standardized ileal digestible.

1 Formulated to meet the nutrient requirements for broilers applicable in the Netherlands (CVB, 2020) but without trace mineral supplementation.

2 Reduced in Ca and dig P vs. CON1 (to account for the expected Ca and dig P contribution of the added phytase), without trace mineral supplementation, with exogenous phytase added at 2,000, 1,500 FTU/kg and 1,000 FTU/kg, in starter, grower and (in Exp. 2 only) finisher phases, respectively. (Inclusion rate < 0.006 % in all cases).

3 Added for potential measurements of nutrient digestibility (that were not ultimately undertaken).

4 Supplied per kilogram of diet: vitamin A (retinyl acetate), 10,000 IU; vitamin D3, 2,500 IU; vitamin E (dl-α-tocopherol), 50 IU; vitamin K3 (menadione), 1.5 mg; vitamin B1 (thiamine mononitrate) 2.0 mg; riboflavin, 7.5 mg; D-pantothenic acid, 12 mg; vitamin B6, 3.5 mg; vitamin B12, 0.020 mg; niacin, 35 mg; folic acid, 1.0 mg; biotin, 0.20 mg; choline (choline chloride), 460 mg; I (KI), 0.8 mg.

5 A novel consensus bacterial 6-phytase variant expressed in *Trichoderma reesei.*

In both studies, the basal diets were prepared from one bulk batch of the common, fixed, ingredients (all ingredients except limestone, MCP, titanium dioxide, supplemental phytase and TM-premixes). This bulk batch was then divided into 2 equal portions to which the remaining fixed ingredients for CON1 (limestone, MCP) and CON2 (limestone, MCP, filler and supplemental phytase) were added. Each of these diets was then sub-divided into 6 sub-batches and a different TM premix added to each (including one treatment without TM, as detailed in Table 1). Diets were mixed and pelleted (2.5 mm pellet for starter diets and 3 mm pellet for grower and finisher phase diets; pelleting temperature ≤ 80 °C).

### Measurements and sampling

In both experiments, birds were weighed on a per pen basis at 0, 10, 20 and 35 d of age. Body weight gain (**BWG**) was calculated, for each individual phase (0 to 10, 10 to 20 and 20 to 35 d of age) and cumulatively. Feed intake (**FI**) was determined per pen, corrected for mortality, for each phase. Feed conversion ratios (**FCR**) were calculated with correction for mortality, per phase and cumulatively. Pens were monitored daily for mortality and dead birds were removed and weighed.

In Experiment 2 only, at each of 10 and 20 d of age, 4 birds per pen were euthanized by gasification with CO_2_. Venous blood samples (∼2 ml) were collected from the neck of 2 birds per pen (two samples per bird). Samples were centrifuged at 20 °C with 3500 rpm for 10 minutes and plasma collected and stored at -20 °C until later analysis. Livers were extracted from 2 birds per pen, weighed as two samples and stored frozen at -20 °C. For liver and plasma samples, one sample was sent for laboratory analysis and the other was stored. Tibia bones were collected from 4 birds per pen, pooled and frozen at -20 °C.

All basal and final diets were sampled for the analysis of TM and phytase activity. Basal diets were additionally analyzed for Ca, P and phytate (**IP_6_**).

### Chemical analysis

Thawed tibias were pooled per pen and defatted in 100 % petroleum ether using a Soxhlet apparatus (ThermoFisher Scientific, Roskilde, Denmark) according to a modified version of the method of Watson et al. (2006). The de-fatted bones were dried and the bone ash content in fat-free DM determined as described by Davin et al., (2020). Macrominerals (Ca, P and Mg) and TM (Zn, Fe, Cu and Mn) in feed, tibia ash and liver samples were analyzed by NutriControl analytical solutions (Veghel, the Netherlands) using validated and accredited methods based on inductively coupled plasma-optical emission spectroscopy (**ICP-OES**). The performance characteristics of the methods adhered to European standard NEN-EN 15510:2017 (NEN-EN, 2017). Trace minerals Zn, Fe, Cu, Mn and Se in plasma were analyzed by Laboklin N. V. (Hoensbroek, the Netherlands). Copper, Zn and Fe were analyzed photometrically using a Cobas 8000 modular analyzer with C701 photometric measuring unit (Roche Diagnostics Ltd.) and commercially available test kits. Selenium and Mn were analyzed by atomic absorption spectrometry (AAS) using a ZEEnit 650 P spectrometer (Analytik Jena GmbH). Phytase activities in feed were analyzed by Danisco Animal Nutrition Research Centre (Brabrand, Denmark) according to a modified version of the 2000.12 AOAC method (Engelen et al., 2001). For this, one FTU was defined as the quantity of enzyme that released 1 µmol of inorganic orthophosphate from a 0.0051 mol/L sodium phytate substrate per minute at pH 5.5 at 37 °C. Phytate (**IP_6_**) in feed was analyzed at Danisco Animal Nutrition Research Centre (Brabrand, Denmark) using the HPLC method described by Christensen et al. (2020) modified from Skoglund et al. (1998).

### Statistical analysis

A pen was the experimental unit for all growth performance and tibia analyses. A bird was the experimental unit for liver and blood analyses. All data were analyzed by 2-way ANOVA. Treatment was included in the model as a fixed effect, block as a random effect. Treatment means were separated using Tukey's HSD test. All statistical analyses were performed in JMP version 16.0 (SAS Institute, Inc., Cary, NC; JMP, 2022). A *P* value of < 0.05 was considered statistically significant, whereas 0.05 ≤ *P* < 0.1 was considered a tendency.

## Results

### Analyzed nutrients and phytase activity

In both experiments, analyzed Ca and P values in treatment 1 (CON 1) and treatment 7 (CON 2) corresponded well (within 10 %) with formulated values, during all phases (Table 2). Analyzed phytate-P concentrations in the basal diets were close to the formulated levels in both experiments (Table 2). In all treatments and phases, analyzed concentrations of Zn, Mn, Cu and Se were also in close agreement (within 15 %) with expected values, in both experiments (Table 3). Analyzed Fe concentrations were close to expected levels in Experiment 1 during all phases and Experiment 2 during finisher phase but were more variable in starter and grower diets in Experiment 2 (-2 to +41 % vs. calculated values). Phytase activities in the unsupplemented diets were low across both studies, as expected (< 200 FTU/kg in treatments 1 and 2, Table 3), and activities in the phytase-supplemented diets were within acceptable limits to confirm the supplementation of phytase in these treatments.

**Table 3:** Analyzed trace mineral content and phytase activities of the treatment diets in Experiment 1 and 2^1^.

Exp. No.	Trace mineral or phytase	Phase	Treatment No.^1^											
			1	2	3	4	5	6	7	8	9	10	11	12
Experiment 1	Zn, mg/kg	Starter	25	35	45	40	54	35	25	33	43	41	54	37
		Grower	25	33	43	39	54	38	25	33	44	40	53	33
	Fe, mg/kg	Starter	153	160	182	164	183	174	124	120	160	127	135	122
		Grower	148	163	175	162	187	152	102	109	131	115	129	111
	Cu, mg/kg	Starter	6	8	12	8	11	8	5	9	11	9	11	10
		Grower	<5	7	11	7	10	7	<5	7	9	7	10	7
	Mn, mg/kg	Starter	30	40	50	39	51	40	24	32	43	36	37	35
		Grower	31	39	47	35	51	37	23	32	42	44	46	33
	Se, mg/kg	Starter	0.11	0.23	0.24	0.23	0.28	0.27	0.10	0.23	0.23	0.25	0.28	0.22
		Grower	<0.1	0.23	0.24	0.28	0.21	0.34	0.11	0.23	0.24	0.20	0.32	0.22
	Phytase activity, FTU/kg^2^	Starter	117	114	-	-	-	-	1,595	1,580	1,695	1,744	1,692	1,813
		Grower	115	98	-	-	-	-	1,333	1,238	1,696	1,678	1,714	1,342
Experiment														
2	Zn, mg/kg	Starter	27	36	46	42	57	37	27	35	43	44	57	38
		Grower	31	40	48	44	60	39	27	38	47	43	62	41
		Finisher	31	40	52	45	62	40	29	41	53	45	62	40
	Fe, mg/kg	Starter	172	173	190	174	203	174	118	120	137	136	135	117
		Grower	168	178	206	193	221	172	135	140	162	149	158	130
		Finisher	159	169	176	172	178	162	122	123	144	116	146	135
	Cu, mg/kg	Starter	5.8	8	12	8.8	12	8.5	5.7	8.7	10	8.6	12	8.7
		Grower	5	6.1	9.9	7.1	10	7.3	5	6.9	8.9	7.3	10	6.3
		Finisher	5.7	8.6	11	8.8	11	7.5	5	7.1	12	7.7	11	8.4
	Mn, mg/kg	Starter	33	39	47	40	52	39	25	34	43	31	40	34
		Grower	32	41	51	39	50	42	26	35	48	32	47	35
		Finisher	25	34	41	31	61	33	19	27	37	30	36	28
	Se, mg/kg	Starter	0.14	0.23	0.27	0.27	0.3	0.27	0.11	0.21	0.27	0.29	0.3	0.27
		Grower	0.16	0.28	0.28	0.28	0.25	0.24	0.14	0.29	0.29	0.26	0.33	0.34
		Finisher	0.1	-	0.22	0.23	0.25	0.23	0.11	0.23	0.27	0.27	0.26	0.27
	Phytase activity, FTU/kg^2^	Starter	89	150	-	-	-	-	1,812	2,188	2,159	2,073	1,815	2,078
		Grower	182	154	-	-	-	-	1,529	1,643	1,597	1,481	1,264	1,521
		Finisher	152	148	-	-	-	-	1,184	923	1,043	1,079	1,377	1,200

- not determined.

1 Treatment details are provided in Table 1.

2 Phytase activities were not analyzed in treatment diets 3 to 6 because no exogenous phytase was supplemented in these diets; it was expected that the level of phytase in these diets would be similar to treatment diet 2.

### Growth performance – Experiment 1

There were interactions between phytase and TM supplementation on BW, BWG and FI during starter (0 to 10 d of age) and grower (10 to 20 d of age) phases (*P* < 0.001 in all cases; Table 4). At 10 d of age, the BW of birds fed diets supplemented with TM but no phytase (treatments 2 to 6) was higher than that of birds fed diets without added TM or phytase (treatment 1; *P* < 0.05), whereas the BW of birds supplemented with phytase but no TM (treatment 7) was greater than that of birds supplemented with TM but no phytase, regardless of source or dose level (treatments 2 to 6; *P* < 0.05). At 20 d of age the response was slightly different (Table 4): Again, the BW of birds supplemented with TM but no phytase (treatments 2 to 6) was higher than that of birds fed diets without added TM or phytase (treatment 1; *P* < 0.05), whereas the BW of birds supplemented with phytase but no TM (treatment 7) was greater than that of birds supplemented with sulphate-, oxide- or organic-based TM at ‘low’ level (treatments 2, 4 and 6) but not than that of birds supplemented with sulphate- or oxide-based TM at ‘high’ level (treatments 3 and 5). Addition of TM on top of phytase (treatments 8 to 12) did not further increase BW above the level achieved by phytase alone (treatment 7) at either timepoint. At both 10 and 20 d of age, the comparison of treatment means for BW provided some evidence suggesting that, in diets without supplemental phytase, there was an effect of TM dose in which BW was higher (*P* < 0.05) in birds supplemented with sulphate-based TM at a ‘high’ vs. ‘low’ dose, whereas in diets containing added phytase, there was no apparent effect of TM dose level on BW.

**Table 4:** Effect of dietary supplementation with trace minerals (low or high) or phytase or both, on growth performance during 0 to 10 and 10 to 20 d of age; 2-way ANOVA (Experiment 1).

Treatment no.^1^	Phytase (±)^2^	Trace mineral supplementation^3^		BW g/bird^4^	BWG, g/bird	FI, g/bird	FCR, g:g
		Source	Level				
*At 0 to 10 d of age:*							
Treatment means							
1	-	-	-	255^e^	213^e^	218^d^	1.026
2	-	sulphate-based	Low	305^d^	262^d^	263^c^	1.006
3	-	sulphate-based	High	324^bc^	281^bc^	281^ab^	1.003
4	-	oxide-based	Low	317^cd^	274^cd^	274^bc^	1.001
5	-	oxide-based	High	326^bc^	283^abc^	285^ab^	1.005
6	-	organic-based	Low	307^d^	264^d^	265^c^	1.007
7	+	-	-	341^a^	297^a^	293^a^	0.989
8	+	sulphate-based	Low	335^ab^	292^ab^	287^ab^	0.984
9	+	sulphate-based	High	336^ab^	293^ab^	288^ab^	0.984
10	+	oxide-based	Low	335^ab^	292^ab^	286^ab^	0.982
11	+	oxide-based	High	334^ab^	292^b^	286^ab^	0.981
12	+	organic	Low	331^abc^	288^abc^	284^ab^	0.983
Main effects							
Phytase	-			306	263	265	1.008^a^
	+			335	292	287	0.984^b^
Trace minerals		-		298	255	256	1.008^a^
		sulphate-based	Low	320	277	275	0.995^b^
		sulphate-based	High	330	287	285	0.994^b^
		oxide-based	Low	326	283	280	0.992^b^
		oxide-based	High	330	287	285	0.993^b^
		organic	Low	319	276	274	0.995^b^
SEM				3.115	3.158	3.059	0.004
*P*-value, phytase				<0.001	<0.001	<0.001	<0.001
*P*-value trace minerals			<0.001	<0.001	<0.001	<0.001	
*P*-value phytase x trace minerals		<0.001	<0.001	<0.001	0.123		
							
*At 10 to 20 d of age*							
Treatment means							
1	-	-	-	827^e^	573^e^	718^d^	1.253
2	-	sulphate-based	Low	969^d^	659^d^	833^c^	1.263
3	-	sulphate-based	High	1,027^abc^	699^abc^	872^abc^	1.247
4	-	oxide-based	Low	1,000^bcd^	682^bcd^	855^abc^	1.253
5	-	oxide-based	High	1,032^ab^	705^abc^	888^a^	1.259
6	-	organic-based	Low	988^cd^	681^cd^	849^bc^	1.246
7	+	-	-	1,048^a^	707^abc^	885^ab^	1.252
8	+	sulphate-based	Low	1,060^a^	723^a^	891^a^	1.233
9	+	sulphate-based	High	1,049^a^	713^a^	883^b^	1.238
10	+	oxide-based	Low	1,047^a^	712^ab^	883^ab^	1.239
11	+	oxide-based	High	1,045^a^	711^abc^	881^ab^	1.239
12	+	organic	Low	1,047^a^	715^a^	882^ab^	1.233
Main effects							
Phytase	-			974	667	836	1.253^a^
	+			1,049	714	884	1.239^b^
Trace minerals		-		938	640	802	1.252
		sulphate-based	Low	1,015	691	862	1.248
		sulphate-based	High	1,038	706	877	1.242
		oxide-based	Low	1,023	697	869	1.246
		oxide-based	High	1,039	708	884	1.249
		organic	Low	1017	698	865	1.240
SEM				8.491	6.475	8.369	0.006
*P*-value, phytase		<0.001	<0.001	<0.001	<0.001		
*P*-value trace minerals		<0.001	<0.001	<0.001	0.448		
*P*-value phytase x trace minerals		<0.001	<0.001	<0.001	0.326		

^a,b,c,d^Means within each column grouping with uncommon superscripts are significantly different at *P* < 0.05.

1 Treatments 1–6 were based on CON1, treatments 7–12 were based on CON2 (see Table 2).

2 Included at 2,000 FTU/kg and 1,500 FTU/kg in starter and grower phases, respectively.

3 The levels of individual trace minerals added to each treatment diet, and their sources, are given in Table 1.

4 At end of phase.

The interaction effect of supplemental phytase and TM on FI during 0 to 10 and 10 to 20 d of age was such that TM and phytase both (independently) increased feed intake but the improvement with phytase was generally greater than that from supplemental TM alone.

**Fig. 1:**
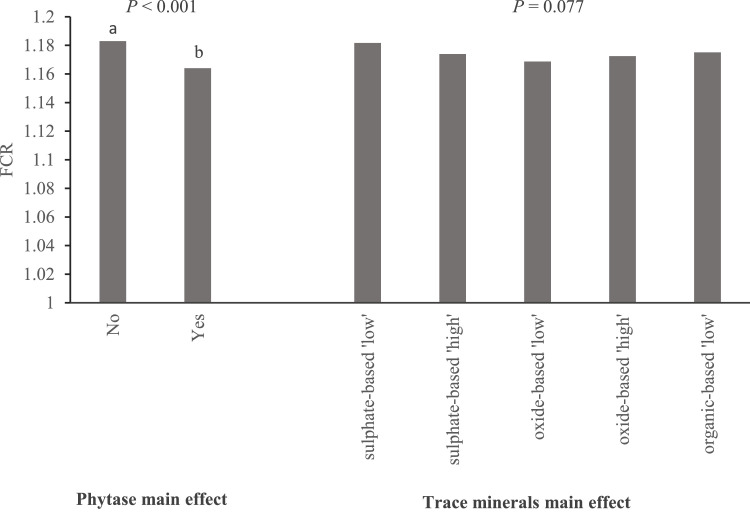
Main effects^1^ of phytase (left hand panel) and trace mineral supplementation^2^ (right hand panel) on FCR during 0–20 d of age in Experiment 1. ^1^2-Way ANOVA, *P*-value, phytase = < 0.001; *P*-value, TM = 0.077; *P*-value, TM x phytase interaction was not statistically significant (*P* = 0.447). ^2^The levels of individual trace minerals added to each treatment diet, and their sources, and of phytase supplementation, are given in Table 1. FCR, feed conversion ratio.

Fig. 1 shows the main effects of phytase and TM supplementation on the FCR response of birds during 0 to 20 d of age; FCR was reduced by phytase (*P* < 0.001) and tended to be reduced by TM supplementation (*P* = 0.077), without interaction. There was no effect of TM source or dose level on FCR at 0 to 20 d of age.

### Growth performance—Experiment 2

During starter phase (0 to 10 d of age; Table 5), there were interactions between supplemental phytase and TM for all response measures (BW, BWG, FI and FCR; *P* < 0.001 in all cases). In the absence of phytase, these measures were consistently improved by TM addition to CON1, regardless of source or dose (treatments 2 to 6 vs. treatment 1; *P* < 0.05); added TM increased BW at 10 d of age by 28 to 37 g/bird vs. no added TM (+10 to +13 % across treatments 2 to 6). Added phytase in CON2 without added TM (treatment 7) also improved these measures, above the level of treatment 1 (*P* < 0.05), not different from treatments 2 to 6 that contained added TM (but no phytase) except for FCR which was improved beyond the level of the low TM supplemented treatments (*P* < 0.05); added phytase in CON2 increased BW at 10 d of age by 32 g/bird vs. no added TM or phytase in CON1 (311 vs. 279 g, +11 %; *P* < 0.05). Supplementation of TM on top of phytase (treatments 8 to 12) did not improve performance above the level of phytase supplementation (treatment 7) except in the ‘low’ dose sulphate- or oxide-based TM treatments [BW, BWG and FI in treatment 10 was higher, and FCR in treatment 8 was lower, than in treatment 7; *P* < 0.05]. The interaction effect of supplemental phytase and TM on FI was similar to that seen in Experiment 1. Growth performance responses did not differ among TM sources or dose levels except for FCR as detailed above.

**Table 5:** Effect of supplementation with trace minerals (‘low’ or ‘high’), phytase, or both, on growth performance during 0 to 10, 10 to 20 and 20 to 35 d of age; 2-way ANOVA (Experiment 2).

Treatment no.^1^	Phytase (±)^2^	Trace mineral supplementation^3^		d 10 BW, g/bird	BWG, g/bird	FI, g/bird	FCR, g:g
		Source	Level				
*At 0 to 10 d of age*							
Treatment means							
1	-	-	-	279^c^	237^e^	245^c^	1.033^a^
2	-	Sulphate-based	Low	308^b^	266^cd^	271^ab^	1.017^b^
3	-	Sulphate-based	High	316^ab^	274^abcd^	276^ab^	1.007^bcde^
4	-	Oxide-based	Low	307^b^	265^d^	268^b^	1.014^bcd^
5	-	Oxide-based	High	313^ab^	271^abcd^	272^ab^	1.003^cdef^
6	-	Organic-based	Low	308^b^	266^d^	270^ab^	1.015^bc^
7	+	-	-	311^b^	269^bcd^	269^b^	1.000^ef^
8	+	Sulphate-based	Low	318^ab^	279^abc^	276^ab^	0.988^g^
9	+	Sulphate-based	High	319^ab^	280^ab^	278^ab^	0.993^f^
10	+	Oxide-based	Low	325^a^	283^a^	282^a^	0.995^ef^
11	+	Oxide-based	High	315^ab^	277^abcd^	277^ab^	1.001^def^
12	+	Organic-based	Low	318^ab^	276^abcd^	276^ab^	0.999^ef^
Main effects							
Phytase	-			305	263	267	1.015
	+			318	277	276	0.996
Trace minerals		-		295	253	257	1.017
		Sulphate-based	Low	313	273	273	1.003
		Sulphate-based	High	318	277	277	1.000
		Oxide-based	Low	316	274	275	1.004
		Oxide-based	High	314	274	275	1.002
		Organic-based	Low	313	271	273	1.007
SEM				3.05	2.55	2.55	0.003
*P*-value, phytase				< 0.001	<0.001	<0.001	<0.001
*P*-value trace minerals			<0.001	<0.001	<0.001	<0.001	
*P*-value phytase x trace minerals		<0.001	<0.001	<0.001	<0.001		
							
*At 10 to 20 d of age*							
Treatment means							
1	-	-	-	937^f^	658^d^	829^d^	1.259
2	-	Sulphate-based	Low	1,017^cde^	706^bc^	886^abc^	1.255
3	-	Sulphate-based	High	1,031^abcde^	715^abc^	894^abc^	1.250
4	-	Oxide-based	Low	1,001^e^	694^c^	867^c^	1.250
5	-	Oxide-based	High	1,023^bcde^	710^abc^	891^abc^	1.255
6	-	Organic-based	Low	1,014^de^	711^abc^	885^abc^	1.246
7	+	-	-	1,033^abcde^	723^ab^	885^abc^	1.224
8	+	Sulphate-based	Low	1,036^abcde^	714^abc^	880^bc^	1.232
9	+	Sulphate-based	High	1,061^a^	736^a^	905^ab^	1.230
10	+	Oxide-based	Low	1,056^ab^	736^a^	914^a^	1.242
11	+	Oxide-based	High	1,051^abc^	736^a^	897^abc^	1.219
12	+	Organic-based	Low	1,045^abcd^	728^ab^	893^abc^	1.227
Main effects							
Phytase	-			1,004	699	875	1.253^a^
	+			1,047	729	896	1.229^b^
Trace minerals	-		985	691	857	1.242	
		Sulphate-based	Low	1,027	710	883	1.243
		Sulphate-based	High	1,046	725	900	1.240
		Oxide-based	Low	1,028	715	891	1.246
		Oxide-based	High	1,037	723	894	1.237
		Organic-based	Low	1,030	719	889	1.236
SEM				7.20	5.73	6.57	0.005
*P*-value, phytase				<0.001	<0.001	<0.001	<0.001
*P*-value trace minerals			<0.001	<0.001	<0.001	0.453	
*P*-value phytase x trace minerals		<0.001	<0.001	<0.001	0.097		
							
*At 20 to 35 d of age:*							
Treatment means							
1	-	-	-	2,471^b^	1,562	2,431	1.558
2	-	Sulphate-based	Low	2,625^a^	1,608	2,521	1.568
3	-	Sulphate-based	High	2,677^a^	1,646	2,538	1.544
4	-	Oxide-based	Low	2,591^ab^	1,591	2,483	1.563
5	-	Oxide-based	High	2,633^a^	1,610	2,496	1.551
6	-	Organic-based	Low	2,640^a^	1,626	2,500	1.538
7	+	-	-	2,645^a^	1,612	2,510	1.557
8	+	Sulphate-based	Low	2,671^a^	1,631	2,500	1.532
9	+	Sulphate-based	High	2,676^a^	1,621	2,473	1.527
10	+	Oxide-based	Low	2,724^a^	1,682	2,593	1.542
11	+	Oxide-based	High	2,694^a^	1,648	2,504	1.520
12	+	Organic-based	Low	2,699^a^	1,654	2,518	1.522
Main effects							
Phytase	-			2,606	1,607^b^	2,495	1.554^a^
	+			2,685	1,641^a^	2,516	1.533^b^
Trace minerals	-		2,558	1,587	2,470	1.558	
		Sulphate-based	Low	2,648	1,620	2,510	1.550
		Sulphate-based	High	2,676	1,633	2,505	1.535
		Oxide-based	Low	2,658	1,636	2,538	1.553
		Oxide-based	High	2,663	1,629	2,500	1.535
		Organic-based	Low	2,670	1,640	2,509	1.530
SEM				29.15	24.48	31.06	0.012
*P*-value, phytase				<0.001	0.017	0.238	0.004
*P*-value trace minerals			0.001	0.295	0.462	0.116	
*P*-value phytase x trace minerals		0.045	0.317	0.074	0.731		

BWG, body weight gain; FI, feed intake; FCR, feed conversion ratio.

^a,b,c,d^Means within each column grouping with uncommon superscripts are significantly different at *P* < 0.05.

1 Treatments 1 to 6 were based on CON1, treatments 7 to 12 were based on CON2 (see Table 2).

2 Included at 2,000, 1,500 and 1,000 FTU/kg in starter, grower and finisher phases, respectively.

3 The levels of individual trace minerals added to each treatment diet, and their sources, are given in Table 1.

During grower phase (d 10 to 20; Table 5), a similar interaction to that seen during starter phase was evident for d 20 BW, BWG and FI (*P* < 0.001 in all cases), and there was a tendency (*P* = 0.097) towards this interaction for FCR. As also seen in starter phase, birds in treatment 7 attained average BW, BWG, FI and FCR values that were not significantly different to those in treatments 2 to 6 and there were no additional improvements when TM were added on top of phytase regardless of TM source or dose level.

During finisher phase (d 20 to 35; Table 5) the response was different. There was an interaction (*P* < 0.05) between phytase and TM only on d 35 BW (and a tendency towards this interaction on FI; *P* = 0.074); TM supplementation had no effect on BWG or FCR. Phytase (main effect) improved both measures (*P* < 0.05) by a lower magnitude than in earlier phases (BWG +2.1 % vs. +5.3 and +4.3 in starter and grower phases, respectively). Growth performance responses did not differ among TM sources or dose levels.

Cumulative growth performance responses during 0 to 20 d of age followed a similar pattern to those seen during grower phases (data not shown). For the overall period (0 to 35 d of age, Table 6) the BWG response also followed a similar pattern, with similar interactions evident (*P* < 0.05); final (d 35) BW, overall BWG, FI and FCR of birds in treatment 7 were improved vs. treatment 1 (*P* < 0.05), not significantly different from treatments 2 to 6 and supplemental TM on top of phytase did not lead to further improvements. Added TM without added phytase increased d 35 BW by 120 to 206 g/bird (+4.9 to 8.3 %; *P* < 0.05) across treatments, vs. no added TM, whereas added phytase without added TM increased BW at 35 d of age by 174 g/bird (2,645 vs 2,471 g, +7.0 %; *P* < 0.05) vs. no added TM or phytase. Supplemental phytase improved (*P* < 0.05) overall 0-35 days of age FCR, some of the TM supplemented treatments (oxide or organic based) improved (*P* < 0.05) overall FCR.

**Table 6:** Effect of dietary supplementation with trace minerals, phytase, or both, on cumulative growth performance during 0 to 35 d of age; 2-way ANOVA (Experiment 2).

Treatment no.^1^	Phytase (±)^2^	Trace mineral supplementation^3^		BWG, g/bird	FI, g/bird	FCR, g:g	Mortality, %
		Source	Level				
Treatment means							
1	-	-	-	2,429^b^	3,476^b^	1.432	0.71
2	-	Sulphate-based	Low	2,583^a^	3,676^a^	1.423	1.79
3	-	Sulphate-based	High	2,635^a^	3,708^a^	1.408	1.43
4	-	Oxide-based	Low	2,549^ab^	3,620^ab^	1.421	0.71
5	-	Oxide-based	High	2,591^a^	3,659^a^	1.413	0.71
6	-	Organic-based	Low	2,598^a^	3,649^ab^	1.405	2.24
7	+	-	-	2,603^a^	3,663^a^	1.407	0.71
8	+	Sulphate-based	Low	2,629^a^	3,661^a^	1.393	2.57
9	+	Sulphate-based	High	2,634^a^	3,653^a^	1.388	1.07
10	+	Oxide-based	Low	2,682^a^	3,764^a^	1.403	1.43
11	+	Oxide-based	High	2,652^a^	3,673^a^	1.385	1.08
12	+	Organic-based	Low	2,657^a^	3,686^a^	1.387	1.07
Main effects							
Phytase	-			2,564	3,632	1.417^a^	1.27
	+			2,643	3,683	1.394^b^	1.32
Trace minerals		-	-	2,516	3,570	1.420^a^	0.71
		Sulphate-based	Low	2,606	3,669	1.408^ab^	2.18
		Sulphate-based	High	2,634	3,681	1.398^ab^	1.25
		Oxide-based	Low	2,616	3,692	1.412^b^	1.07
		Oxide-based	High	2,621	3,666	1.399^b^	0.90
		Organic-based	Low	2,628	3,668	1.396^b^	1.65
SEM				29.14	36.50	0.007	0.703
*P*-value, phytase				<.0001	0.016	<.0001	0.886
*P*-value, trace minerals			0.001	0.018	0.004	0.326	
*P*-value, phytase x trace minerals		0.045	0.009	0.905	0.737		

BWG, body weight gain; FI, feed intake; FCR, feed conversion ratio.

n.d., not determined.

^a,b,c,d^Means within each column grouping with uncommon superscripts are significantly different at *P* < 0.05.

1 Treatments 1 to 6 were based on CON1, treatments 7 to 12 were based on CON2 (see Table 2).

2 Included at 2,000, 1,500 and 1,000 FTU/kg in starter, grower and finisher phases, respectively.

3 The levels of individual trace minerals added to each treatment diet, and their sources, are given in Table 1.

Overall, mortality was consistently low (<2.6 % among treatments, 1.3 % on average) and unaffected by phytase or TM supplementation (Table 6).

### Bone mineralization—Experiment 2

At 10 d of age (Table 7), there were interactions between TM and phytase for tibia ash (*P* < 0.05), tibia concentrations of Zn (*P* < 0.001) and Mn (*P* < 0.05), and a tendency towards an interaction for tibia Fe (*P* = 0.080). Without phytase, tibia ash was increased by TM supplemented at ‘high’ level (treatments 3 and 5 vs. 1; *P* < 0.05), whereas addition of phytase without TM to the Ca and P-reduced CON2 diet (treatment 7 vs. 1) or addition of TM on top of phytase (treatments 8 to 12 vs. 7) maintained tibia ash equivalent to nutritionally adequate treatment 1. In the absence of phytase, added TM markedly increased tibia Zn (+105 to 193 mg/kg DM or +64 to 117 % in treatments 2–6 vs. 1; *P* < 0.05), with greater response levels (*P* < 0.05) in TM ‘high’ than TM ‘low’ treatments and in oxide- vs. sulphate- or organic-based TM treatments (*P* < 0.05). Added phytase without added TM markedly increased tibia Zn compared to either no added phytase or TM (treatment 1) or to added TM but no phytase (treatments 2 to 6); the effect sizes were +228 mg/kg DM, equivalent to +138 % in treatment 7 vs. 1 (*P* < 0.05) and +35 to + 123 mg/kg DM, equivalent to +10 to +46 %, in treatment 7 vs. 2 to 6; *P* < 0.05). Addition of TM on top of phytase did not further increase tibia Zn. In contrast, tibia Mn was not increased by TM supplementation without phytase, except in treatment 5 (oxide-based ‘high’), whereas phytase in CON2 markedly increased tibia Mn (+29 % in treatment 7 vs. 1; *P* < 0.05). Added TM on top of phytase further improved tibia Mn above the level achieved by phytase alone, regardless of TM source or dose-level (+24 to +44 % in treatments 8 to 12 vs. 7; *P* < 0.05). Tibia Fe was moderately (-4.4 to -7.2 %) but significantly (*P* < 0.05) reduced by supplemental TM in all but one TM treatment (sulphate-based ‘low’ TM) but was increased by phytase (+7 % vs. no phytase; *P* < 0.05). Tibia Cu was not increased by TM or phytase and in fact tended (*P* = 0.079) to be reduced by phytase (-14 % vs. no phytase). Tibia Ca and P in birds fed the phytase-supplemented CON2 diet (treatments 7 to 12) were maintained at a level that was not significantly different from that of birds fed the nutritionally adequate CON1 diet (treatment 1) and there was no effect of TM supplementation. Tibia Mg was increased by phytase (+2.8 % vs no phytase; *P* < 0.05).

**Table 7:** Effect of dietary supplementation with trace minerals, phytase, or both, on tibia ash, mineral and trace mineral content at 10 and 20 d of age; 2-way ANOVA (Experiment 2).

Treatment no.^1^	Phytase (±)^2^	Trace mineral supplementation^3^		Tibia ash, g/kg fat-free DM	Zn, mg/kg DM	Fe, mg/kg DM	Cu, mg/kg DM	Mn, mg/kg DM	Ca, g/kg DM	P, g/kg DM	Mg, g/kg DM
		Source	Level								
*At 10 d of age*											
Treatment means											
1	-	-	-	493^bc^	165^f^	249	4.82	4.79^fg^	354	176	8.90
2	-	Sulphate-based	Low	495^abc^	270^e^	233	3.76	5.00^efg^	354	179	9.01
3	-	Sulphate-based	High	501^a^	324^c^	219	2.91	5.66^def^	352	178	9.17
4	-	Oxide-based	Low	494^abc^	304^d^	224	3.47	4.86^g^	354	176	9.21
5	-	Oxide-based	High	500^a^	358^b^	230	3.88	5.72^de^	353	174	8.97
6	-	Organic-based	Low	492^bc^	278^e^	224	3.56	4.72^g^	351	178	9.18
7	+	-	-	492^bc^	393^a^	248	3.56	6.16^d^	351	179	9.19
8	+	Sulphate-based	Low	490^c^	397^a^	249	2.67	8.09^abc^	353	179	9.40
9	+	Sulphate-based	High	496^abc^	399^a^	246	3.54	8.84^a^	351	176	9.28
10	+	Oxide-based	Low	491^bc^	394^a^	250	2.94	7.84^bc^	353	180	9.17
11	+	Oxide-based	High	489^c^	404^a^	245	3.27	8.54^ab^	352	179	9.51
12	+	Organic-based	Low	497^ab^	396^a^	237	3.16	7.62^c^	351	178	9.38
Main effects											
Phytase	*-*			496	283	230^b^	3.73	5.12	353	177	9.07^b^
	*+*			493	397	246^a^	3.19	7.85	352	179	9.32^a^
Trace minerals	-		493	279	249^a^	4.19	5.47	352	178	9.05	
		Sulphate-based	Low	493	334	241^ab^	3.22	6.54	353	179	9.20
		Sulphate-based	High	498	362	233^bc^	3.23	7.25	351	177	9.23
		Oxide-based	Low	493	348	237^bc^	3.21	6.35	353	178	9.19
		Oxide-based	High	495	381	238^bc^	3.58	7.13	352	177	9.24
		Organic-based	Low	495	337	231^c^	3.36	6.17	351	178	9.28
SEM				2.432	6.233	5.003	0.529	0.290	2.651	2.533	0.113
*P*-value, phytase			0.029	<0.001	<0.001	0.079	<0.001	0.441	0.277	<0.001	
*P*-value trace minerals			0.170	<0.001	0.008	0.388	<0.001	0.896	0.922	0.423	
*P*-value phytase x trace minerals		0.040	<0.001	0.080	0.568	0.039	0.999	0.731	0.153		
											
*At 20 d of age*											
Treatment means											
1	-	-	-	527	170^f^	258	2.91	5.73	369	181	7.51
2	-	Sulphate-based	Low	523	241^e^	261	3.03	5.90	369	181	7.43
3	-	Sulphate-based	High	523	271^c^	243	2.73	6.11	368	180	7.38
4	-	Oxide-based	Low	526	265^cd^	243	2.30	6.36	368	181	7.47
5	-	Oxide-based	High	525	282^bc^	255	2.75	6.47	368	178	7.54
6	-	Organic-based	Low	526	249^de^	251	2.66	6.21	367	181	7.45
7	+	-	-	521	300^a^	254	2.72	6.46	368	180	7.49
8	+	Sulphate-based	Low	520	296^ab^	263	2.47	7.73	368	181	7.61
9	+	Sulphate-based	High	521	293^ab^	240	2.36	8.21	367	179	7.67
10	+	Oxide-based	Low	518	291^ab^	269	2.54	7.61	368	181	7.74
11	+	Oxide-based	High	516	303^a^	259	2.28	7.84	369	180	7.82
12	+	Organic-based	Low	518	296^ab^	247	2.81	7.68	367	180	7.63
Main effects											
Phytase	-			524^a^	246	252	2.73	6.13^b^	368	180	7.46^b^
	+			519^b^	297	255	2.53	7.59^a^	368	180	7.66^a^
Trace minerals	-		524	236	256	2.81	6.10^b^	368	180	7.5	
		Sulphate-based	Low	522	269	262	2.75	6.82^a^	368	181	7.5
		Sulphate-based	High	522	282	242	2.54	7.16^a^	367	180	7.5
		Oxide-based	Low	522	278	256	2.42	6.98^a^	368	181	7.6
		Oxide-based	High	520	293	257	2.55	7.16^a^	368	179	7.7
		Organic-based	Low	522	272	249	2.73	6.94^a^	367	181	7.5
SEM				2.411	3.821	11.426	0.306	0.222^a^	1.330	1.936	0.098
*P*-value, phytase			<0.001	<0.001	0.602	0.271	<0.001	0.581	0.897	0.001	
*P*-value trace minerals			0.698	<0.001	0.591	0.765	<0.001	0.920	0.887	0.422	
*P*-value phytase x trace minerals		0.529	<0.001	0.774	0.716	0.060	0.989	0.970	0.629		
											

^a,b,c,d^Means within each column grouping with uncommon superscripts are significantly different at *P* < 0.05.

1 Treatments 1–6 were based on CON1, treatments 7 to 12 were based on CON2 (see Table 2).

2 Included at 2,000, 1,500 and 1,000 FTU/kg in starter (d 0 to 10), grower (d 10 to 20) and finisher (d 20 to 35) phases, respectively.

3 The levels of individual trace minerals added to each treatment diet, and their sources, are given in Table 1.

At 20 d of age (Table 7), there was no interaction between TM and phytase for tibia ash. Tibia ash was unaffected by TM supplementation but was slightly reduced by phytase (519 vs. 524 g/kg, or -1.0 %; *P* < 0.05). A similar interaction to that seen at 10 d of age was evident for tibia Zn concentrations (*P* < 0.001) but with slightly lower effect sizes (+71 to 112 mg/kg or +42 to 66 % in treatments 2 to 6 vs. treatment 1; *P* < 0.05) whereas phytase in treatment 7 increased tibia Zn by 130 mg/kg vs. treatment 1 (+77 %; *P* < 0.05) and by 18 to 59 mg/kg vs. treatments 2 to 6 (+7 to 24 %; *P* < 0.05). Again, there was no further increase in tibia Zn when TM were added on top of phytase (vs. phytase alone). Unlike at 10 d of age, at 20 d of age tibia Mn was increased by supplemental TM (any source or dose level; *P* < 0.001) and by phytase (*P* < 0.001) and adding TM to diets already containing phytase tended to further increase tibia Mn (interaction *P* = 0.060). As at 10 d of age, tibia Mg at 20 d of age was increased by phytase (*P* < 0.001) and tibia Ca and P were maintained by phytase in CON2 at levels that were not significantly different from nutritionally adequate CON1 (treatment 1).

### Trace minerals in the liver—Experiment 2

At 10 d of age (Table 8), there was no effect of phytase or TM supplementation on liver weight as a percentage of BW but supplemental phytase increased absolute liver weights (g) by 5.7 % (phytase main effect; *P* < 0.05). There was an interaction effect on liver concentrations of Zn (*P* < 0.01): Added TM without phytase (in CON1) numerically increased liver Zn (by 4.5 to 10.9 % in treatments 2 to 6 vs. 1; *P >* 0.05), whereas added phytase without added TM in CON2 (treatment 7) significantly increased (*P* < 0.05) liver Zn (+2.7 mg/kg DM or 17 % vs. treatment 1) and TM supplementation on top of phytase numerically reduced liver Zn (treatments 8 to 12 vs. 7; n.s.), regardless of TM source or dose. Liver Fe was increased by phytase (+26 mg/kg or 42 % vs. no phytase; *P* < 0.001) and was higher in sulphate-based ‘high’ than oxide- or organic-based ‘low’ treatments (*P* < 0.05) but the addition of TM without phytase in treatments 2–6 did not increase liver Fe vs. no TM or phytase in treatment 1. Liver Cu concentrations were unaffected by supplemental TM but were reduced by supplemental phytase (-0.09 mg/kg or -3.5 % vs. no phytase; *P* < 0.05). Supplemental phytase increased liver Mn (+0.34 mg/kg or +19 % vs. no phytase; *P* < 0.05) and, to a lesser degree and only in certain treatments, supplemental TM also increased liver Mn (+0.13 to +0.31 mg/kg or +7.4 to +18 % vs. no added TM; Table 8).

**Table 8:** Effect of dietary supplementation with trace minerals, phytase, or both, on liver trace mineral content at 10 and 20 d of age; 2-way ANOVA (Experiment 2).

Treatment no.^1^	Phytase (±)^2^	Trace mineral supplementation^3^		Liver weight, g	Liver weight, % BW	Zn, mg/kg	Fe, mg/kg	Cu, mg/kg	Mn, mg/kg
		Source	Level						
*At 10 d of age:*									
Treatment means									
1	-	-	-	23.0	3.96	15.6^c^	57.5	2.49	1.60
2	-	Sulphate-based	Low	23.3	3.83	16.3^bc^	65.0	2.54	1.85
3	-	Sulphate-based	High	25.1	3.86	17.1^abc^	77.5	2.60	1.90
4	-	Oxide-based	Low	24.9	3.97	16.8^abc^	56.9	2.65	1.77
5	-	Oxide-based	High	25.2	3.93	17.3^abc^	62.9	2.54	1.88
6	-	Organic-based	Low	24.8	3.95	16.6^abc^	48.9	2.61	1.64
7	+	-	-	24.3	3.86	18.3^a^	86.0	2.53	1.91
8	+	Sulphate-based	Low	26.6	4.00	17.4^ab^	92.0	2.50	2.14
9	+	Sulphate-based	High	25.2	4.00	17.1^abc^	93.5	2.50	2.24
10	+	Oxide-based	Low	26.8	3.96	17.0^abc^	70.7	2.41	2.03
11	+	Oxide-based	High	26.2	4.05	17.4^ab^	96.9	2.49	2.23
12	+	Organic-based	Low	25.7	3.93	17.3^abc^	84.1	2.48	2.15
Main effects									
Phytase	-			24.4^b^	3.92	16.6	61.4^b^	2.57^a^	1.77^a^
	+			25.8^a^	3.97	17.4	87.2^a^	2.48^b^	2.11^b^
Trace minerals		-		23.6	3.91	16.9	71.8^ab^	2.51	1.76^b^
		Sulphate-based	Low	25.0	3.92	16.8	78.5^ab^	2.52	1.99^a^
		Sulphate-based	High	25.1	3.93	17.1	85.5^a^	2.55	2.07^a^
		Oxide-based	Low	25.9	3.97	16.9	63.8^b^	2.53	1.90^ab^
		Oxide-based	High	25.7	3.99	17.3	79.9^ab^	2.51	2.05^a^
		Organic-based	Low	25.3	3.94	16.9	66.5^b^	2.54	1.89^ab^
SEM				1.00	0.10	0.37	6.40	0.07	0.06
*P*-value, phytase		0.017	0.389	<0.001	<0.001	0.023	<0.001		
*P*-value trace minerals		0.294	0.964	0.767	0.007	0.981	<0.001		
*P*-value phytase x trace minerals		0.692	0.725	0.005	0.432	0.398	0.403		
									
*At 20 d of age:*									
Treatment means									
1	-	-	-	61.6	3.14	16.5^b^	73.1^b^	2.23	1.86
2	-	Sulphate-based	Low	64.8	3.14	17.8^ab^	80.7^ab^	2.36	2.05
3	-	Sulphate-based	High	65.3	3.14	18.4^a^	76.1^b^	2.39	2.06
4	-	Oxide-based	Low	62.6	3.10	17.6^ab^	93.4^ab^	2.25	2.11
5	-	Oxide-based	High	62.2	3.04	18.0^ab^	80.6^ab^	2.39	2.21
6	-	Organic-based	Low	67.1	3.18	17.8^ab^	76.1^b^	2.41	1.99
7	+	-	-	61.6	3.08	18.8^a^	113.1^a^	2.44	2.25
8	+	Sulphate-based	Low	66.3	3.10	18.3^a^	97.1^ab^	2.44	2.20
9	+	Sulphate-based	High	69.2	3.15	18.0^ab^	110.9^a^	2.46	2.36
10	+	Oxide-based	Low	67.2	3.05	18.1^ab^	85.7^ab^	2.39	2.29
11	+	Oxide-based	High	69.2	3.13	18.7^a^	98.9^ab^	2.49	2.43
12	+	Organic-based	Low	64.8	3.02	18.6^a^	113.0^a^	2.43	2.43
Main effects									
Phytase	-			63.9^b^	3.12	17.7	80.0	2.34^b^	2.05^b^
	+			66.4^a^	3.09	18.4	103.1	2.44^a^	2.33^a^
Trace minerals		-		61.6^b^	3.11	17.6	93.1	2.33	2.06^b^
		Sulphate-based	Low	65.6^ab^	3.12	18.0	88.9	2.40	2.13^ab^
		Sulphate-based	High	67.2^a^	3.14	18.2	93.5	2.43	2.21^ab^
		Oxide-based	Low	64.9^ab^	3.08	17.9	89.5	2.32	2.20^ab^
		Oxide-based	High	65.7^ab^	3.08	18.4	89.7	2.44	2.32^a^
		Organic-based	Low	66.0^ab^	3.10	18.2	94.6	2.42	2.21^ab^
SEM				1.867	0.066	0.366	7.381	0.063	0.088
*P*-value, phytase				0.0252	0.356	0.001	<0.001	0.006	<0.001
*P*-value trace minerals			0.0752	0.925	0.407	0.954	0.263	0.077	
*P*-value phytase x trace minerals		0.1598	0.555	0.025	0.017	0.710	0.476		

^a,b,c,d^Means within each column grouping with uncommon superscripts are significantly different at *P* < 0.05.

1 Treatments 1 to 6 based on CON1, treatments 7 to 12 based on CON2 (see Table 2).

2 Included at 2,000, 1,500 and 1,000 FTU/kg in starter, grower and finisher phases, respectively.

3 The levels of individual trace minerals added to each treatment diet, and their sources, are given in Table 1.

At 20 d of age (Table 8), effects on liver Zn and Mn were similar to those at 10 d of age but with slightly smaller effect sizes (liver Zn + 2.3 mg/kg DM or 14 % in treatment 7 vs. 1; *P* < 0.05). For liver Fe, there was an interaction where added TM did not increase liver Fe when phytase was absent but added phytase without added TM markedly increased liver Fe (+40 mg/kg or +55 % in treatment 7 vs. 1; *P* < 0.05), with no further increase from TM addition on top of phytase. Unlike at 10 d of age, at 20 d of age liver Cu was increased by phytase (+0.1 mg/kg or +4.3 % vs. no phytase).

### Trace minerals in the plasma—Experiment 2

There was an interaction between phytase and TM supplementation on plasma Zn concentrations at 20 d of age (*P* < 0.01; Table 9). Added TM in CON1 (without phytase) increased plasma Zn in all but one treatment (+3.9 to +4.9 µmol/L or +23 to +29 % across treatments 2, 3, 5 and 6 vs. 1; *P* < 0.05) and added phytase (in CON2) without TM also increased plasma Zn (+5.9 µmol/L or +35 % in treatment 7 vs. 1; *P* < 0.05), but TM supplementation on top of phytase in CON2 did not further increase plasma Zn. Plasma concentrations of Fe, Cu and Mn were unaffected by phytase or TM supplementation. For plasma Se, there was a significant interaction (*P* < 0.05); plasma Se was markedly increased by TM supplementation, but the increases were lower in diets already containing phytase (+9 to 11-fold in treatments 2 to 6 vs. treatment 1; +6–8-fold in treatments 8 to 12 vs treatment 7; *P* < 0.05). Plasma Se was not increased by phytase in CON2 vs. CON1 without added phytase or TM. There was no effect of TM source or dose level on plasma concentrations of Zn, Cu, Fe or Mn.

**Table 9:** Effect of dietary supplementation with trace minerals, phytase, or both, on plasma trace mineral content at 20 d of age; 2-way ANOVA (Experiment 2).

Treatment no.^1^	Phytase (±)^2^	Trace mineral supplementation^3^		Zn, µmol/L	Fe, µmol/L	Cu, µmol/L	Mn, µg/L	Se, µg/L
		Source	Level					
Treatment means								
1	-	-	-	16.95^b^	15.00	1.23	47.73	17.3^b^
2	-	Sulphate-based	Low	20.86^a^	15.33	1.08	34.98	171.9^a^
3	-	Sulphate-based	High	21.06^a^	13.95	0.70	37.54	148.2^a^
4	-	Oxide-based	Low	20.54^ab^	13.75	0.65	38.63	157.7^a^
5	-	Oxide-based	High	21.70^a^	13.61	0.64	27.13	161.0^a^
6	-	Organic-based	Low	21.85^a^	15.40	0.94	36.21	188.1^a^
7	+	-	-	22.81^a^	15.35	0.79	29.67	22.9^b^
8	+	Sulphate-based	Low	21.06^a^	13.64	0.61	38.22	172.2^a^
9	+	Sulphate-based	High	21.51^a^	14.25	0.84	32.48	183.7^a^
10	+	Oxide-based	Low	21.16^a^	14.56	0.69	34.93	149.8^a^
11	+	Oxide-based	High	22.65^a^	14.31	0.85	26.14	189.5^a^
12	+	Organic-based	Low	20.38^ab^	14.26	0.70	34.67	146.5^a^
Main effects								
Phytase	-			20.49	14.51	0.87	37.04	140.7
	+			21.60	14.40	0.75	32.68	144.1
Trace minerals		-		19.88	15.18	1.01	38.70	20.1
		Sulphate-based	Low	20.96	14.48	0.84	36.60	172.1
		Sulphate-based	High	21.29	14.10	0.77	35.01	165.9
		Oxide-based	Low	20.85	14.16	0.67	36.78	153.7
		Oxide-based	High	22.18	13.96	0.74	26.64	175.2
		Organic-based	Low	21.11	14.83	0.82	35.44	167.3
SEM				0.757	0.741	0.152	10.554	12.51
*P*-value, phytase				0.014	0.797	0.157	0.477	0.640
*P*-value trace minerals			0.101	0.543	0.343	0.895	<0.001	
*P*-value phytase x trace minerals		0.002	0.430	0.104	0.942	0.037		

^a,b,c,d^Means within each column grouping with uncommon superscripts are significantly different at *P* < 0.05.

1 Treatments 1 to 6 based on CON1, treatments 7 to 12 based on CON2 (see Table 2).

l Included at 2,000, 1,500 and 1,000 FTU/kg in starter, grower and finisher phases, respectively.

3 The levels of individual trace minerals added to each treatment diet, and their sources, are given in Table 1.

## Discussion

In both experiments, the diet analyses indicated that phytase was present in the supplemented treatments at approximately the target dose levels. The intended nutrient and TM levels were also (approximately) achieved with the exception that Fe levels in starter and grower phase basal diets [treatment 1 (CON1) and treatment 7 (CON2)] were higher than expected, especially in Experiment 2. This could have been due to the coccidiostat ingredient (sacox) that was present in starter and grower diets only and had a high Fe content (412 mg/kg, analyzed value). As Fe levels in the basal (unsupplemented) diets were already considerably above the NRC (1994) recommendation of 80 mg/kg, this could have reduced the impact of the added Fe on bird responses during these phases. However, it is unlikely to have impacted on health as an effect on health is not generally seen below a level of 800 mg Fe/kg (Han et al., 2022).

The impaired growth of birds fed the CON1 diet without added TM or phytase, that was observed during starter phase in both experiments, suggests that the bioavailability of TM in the unsupplemented diet was insufficient to meet requirements for growth. This is consistent with the diet analysis which showed that the basal diet was deficient (vs. NRC, 1994 recommendations) in Zn, Cu and Mn, and with previous studies showing that TM withdrawal from 1 d of age, particularly withdrawal of Zn, (in diets without supplemental phytase), resulted in poor growth and leg problems (Bao et al., 2007; Bao and Choct, 2009; Bao et al., 2010). No leg problems were observed in our study. However, tibia concentrations of Zn at 10 and 20 d of age and Mn at 20 d of age were reduced in birds fed CON1 compared to CON1 plus TM, suggesting TM deficiency. Considering the actual growth performance of birds fed the ‘high’ TM treatments without added phytase (treatments 3 and 5), average BW at 21 d of age in both experiments exceeded the breeder performance objective (objective at 21 d of age = 1,012 g/bird; achieved in experiment 1: 1,027 (treatment 3), 1,032 (treatment 5) and in experiment 2: 1,023 (treatment 3), 1,031 (treatment 5). This implies that the TM provided in treatment diets 3 and 5 were sufficient to achieve normal growth performance (BW) for the breed. During finisher phase, the growth performance data provided no evidence of TM deficiency in the unsupplemented diet. Again, this is consistent with earlier studies that have reported no adverse effect of TM withdrawal from 21 d of age or later (Skinner et al., 1992; Christmas et al., 1995). The higher demand of younger birds for TM to support bone development and other key physiological processes explains these differential effects by phase.

Supplementation of TM to CON1 (without added phytase) improved growth performance during starter and grower phases in both experiments. Some of this improvement may have come from the increased feed (and consequently nutrient and energy) intake that was evident. Reported effects of TM supplementation or withdrawal on feed intake in other broiler studies have been mixed. Some studies reported no effect (Franklin et al., 2021; Maiorka et al., 2002) and others reported an increase (Bao et al., 2010). A meta-analysis of 30 articles revealed that Zn supplementation significantly increased feed intake in broilers (Ogbuewu et al., 2023). The authors suggested that the mechanism by which Zn regulates feed intake in chicken is not clear but could involve Zn blocking the release of cholecystokinin from the small intestine. The improved weight gain from TM supplementation is reasoned to have been a direct effect of the increased feed intake and the increased TM availability. Increased TM bioavailability in the TM-supplemented treatments was suggested in Experiment 2 by increased TM concentrations in the tibia ash, liver or plasma but this was only for some individual TM (notably Zn, Mn and Se in plasma); the effects of TM supplementation at the level of these tissues were complex and could be related to the content of these trace minerals in the basal diets and absorption capacity of these TM by the birds. Interestingly, supplemental Se markedly increased plasma Se, more so than the effect on other TM. A similar observation was made by Wang et al. (2022) who reported a quadratic increase in plasma Se from supplemental Se (also as selenite) in the range of 0.10 to 0.50 mg Se/kg. The reason for the greater effect of TM supplementation on plasma Se than on plasma concentrations of other TM is unclear but may be because inorganic Se is readily absorbed by the body via simple diffusion and, unlike for other TM, absorption is largely unaffected by Se status (Lei et al., 2022). In addition the low level of Se content in the basal diets may explain the higher increase in Se in the plasma in the TM supplemented treatments.

Supplemental phytase without supplemental TM in CON2 improved growth performance, to a similar extent as TM supplementation in CON1, suggesting that the phytase replaced the beneficial effect of added TM on performance. Since CON2 was reduced in Ca and retainable P and CON1 was not, it could be argued that this effect was due to reduced antagonism between TM and Ca or P at sites of absorption; interactions between Ca and TM levels in the diet on performance have been observed (Faria et al., 2020) where a high (12 g/kg) or low (8 g/kg) level of Ca reduced the beneficial effect of supplemental TM on performance. However, it is well documented that PhyG improves the availability of both Ca and P in broilers (Babatunde et al., 2021, 2022; Dersjant-Li et al., 2020), confirmed in the present study by the fact that tibia P and Ca concentrations were unaffected by treatment even though CON2 was reduced in Ca and P vs. CON1. The similar growth performance response to phytase supplementation observed in experiments 1 and 2 that were carried out with different Ca levels in the basal diets, further suggests that Ca was not a major factor in improving (or reducing) TM availability in the phytase-supplemented diets.

The improved FI in the phytase supplemented diets without added TM is considered to have been due to the degradation of phytate and consequential effects. The nutrients liberated by phytate degradation may stimulate the expression of appetitive hormones (Kriseldi et al., 2021). Increased feed intake in phytase-supplemented broilers has been observed on previous occasions, including from PhyG phytase, but effects appear to be dose-dependent and also related to the P deficiency of the diet (Dersjant-Li et al., 2022c; Bello et al., 2022). In the present study, the improvements in growth performance from phytase were not only due to increased FI because FCR was also improved, the latter implying improved feed efficiency.

A beneficial effect of PhyG on TM can be explained by its capacity to rapidly and extensively hydrolyze phytate in the low pH conditions of the upper GIT (Christensen et al., 2020; Dersjant-Li et al., 2022b). This is expected to reduce TM binding with phytate and thereby improve TM availability from the basal ingredients for absorption and utilization for growth. The degree of improvement in performance effected by the supplemental phytase in CON2 (without supplemental TM) was, in both studies and for all measures, not significantly different from or greater than that achieved by TM supplementation in CON1 (regardless of TM source or dose level). The implication is that the phytase totally replaced the beneficial effect of the added Zn, Cu, Fe and Mn at ‘high’ or ‘low’ level, in diets with differing Ca levels. The TM content in the basal diets was below NRC recommendation but the NRC recommendation is based on total TM content, not available TM content. This study has demonstrated that by improving the bioavailability of TM in the basal ingredients of the diet, supplementatal phytase increased the availability of TM to levels that met bird requirements. The growth performance outcomes achieved by birds fed the CON2 diets containing added phytase but no added TM were close to, or above, breeder objectives [d 35 BW: 109 % of objective (2,645 g/bird vs. 2,441 g/bird); d 1 to 35 FCR: 99 % of objective (1.407 vs. 1.390); Aviagen Inc., 2022], further indicating that nutrient requirements were met by this diet. In addition, TM supplementation of CON2 did not result in any consistent improvement above that achieved by CON2 without TM suggesting that TM availability in CON2 with phytase was sufficient. [In one treatment (oxide-based ‘low’ TM during 1 to 0 d of age in Experiment 2) there was a performance (BWG, FI and FCR) improvement from adding TM beyond that achieved by the supplemental phytase but as this was not evident in the ‘high’ oxide-based TM treatment and was not matched by any increase in tibia or liver TM, it is unclear that this was due to the added TM].

To the authors’ knowledge, this is the first report of total replacement of the beneficial effects of supplemental TM on growth performance by an exogenous phytase. Comparable studies are scarce. Shelton and Southern (2006) tested the effect of an *Aspergillus niger* phytase added to broiler diets at 600 FTU/kg with, or without, TM supplementation, but did not report any reduction in performance from TM-withdrawal (over 43 d) and the phytase improved performance equally in TM supplemented and unsupplemented diets so its TM-replacement capacity was unclear. The different TM content of the basal diet compared to the present study may explain the different result. In the present study, the basal diet contained low Zn, Mn and Cu content (below NRC, 1994 recommendations), making it more likely that TM and phytase supplementation would have a beneficial effect, whereas in Shelton and Southern (2006) they were adequate in Zn (above the NRC, 1994 recommendation, other TM were not analyzed). In another study, Jondreville et al. (2007) observed improved BWG during 0 to 20 d of age in broilers fed diets containing 12 mg/kg of supplemental Zn but no independent improvement from an *Aspergillus niger* phytase dosed at 280 to 850 FTU/kg. The low native Zn content of the basal diet [33 mg/kg, below the NRC (1994) recommendation of 40 mg/kg] may explain why improved BWG was observed when TM were supplemented, similar to the present study, whereas the different effect of the phytase (absence of an effect) could be due to the dose used or a difference in its specific biochemical and enzymatic properties vs. PhyG. Such differences lead to variation between phytases in their capacity to break down phytate in the early GIT (Menezes-Blackburn et al., 2015) and therefore could affect their efficacy to reduce TM-phytate binding.

Broiler requirements for individual TM by age have yet to be fully defined, mainly due to a lack of research and standardized methodologies for determining TM bioavailability in practical diets (Oviedo-Rondón, 2012; Navidshad et al., 2019). Hence, in commercial diets formulated levels of TM are usually maintained equal across all phases. However, our findings showed that the improvements in BW that were observed in TM-supplemented diets without added phytase were not equal across phases but declined with age. Skinner et al. (1992) and Christmas et al. (1995) made similar observations. It may have been that the TM requirement was lower in finisher phase or that utilization of TM became more efficient as the birds matured. Either way, our results indicate that in diets without added phytase, it may be unnecessary to supplement TM during finisher phase to achieve normal growth performance, whereas in diets containing the added phytase, it may be unnecessary to supplement TM during any phase.

There was little effect of supplemental TM or phytase on tibia ash (Experiment 2). The small (5 g/kg CM or 1.0 % on average) reduction at 20 d of age in treatments supplemented with phytase (vs. no phytase) was unexpected but not evident at 10 d of age and not accompanied by any reduction in tibia Ca or P (despite the mineral matrix applied), so does not suggest that bone mineralization was impaired. On the contrary, the tibia content of several individual TM and of Mg was increased by phytase. The effect was most marked for Zn and Mn which may have been because these TM content were low in the basal diets (lower than NRC recommendations) and because Zn is the first limiting TM in poultry (Bao et al., 2010) and has a higher affinity than other TM cations to bind with phytate (Maddaiah et al., 1964; Persson et al., 1998). The degradation of phytate by phytase would therefore be expected to improve the availability of Zn more substantially than other TM. Tibia Zn levels were markedly increased at both 10 and 20 d of age by supplemental TM and by supplemental phytase without supplemental TM, but the increase from phytase was greater. Shelton and Southern (2006) similarly observed increased tibia Zn concentrations from supplemental TM and from phytase, although not up to the level of the control (TM-supplemented) diet. Meanwhile, Sebastian et al. (1996) observed no effect of supplemental phytase on tibia Zn or other TM, which again may be linked to the TM content of the basal diet. In the present study, supplementation of TM on top of phytase did not further increase tibia Zn, but did further increase tibia Mn, at both 10 and 20 d of age. This may imply that bird requirements for Mn in bone were not maximized by the supplemental phytase without additional TM, even though growth performance outcomes were similar.

We observed a negative effect of supplemental TM on tibia Fe levels at 10 but not 20 d of age. This response is difficult to interpret, and to our knowledge has not been reported previously. It may have been influenced by the high analyzed Fe content of the CON1 basal diet which may have lessened the beneficial effect of supplemental Fe on its utilization in bone. It could also indicate that when TM were supplemented (without phytase) the balance of available TM in the gut promoted uptake and utilization in bone of other TM over Fe, whereas when phytase was supplemented without TM the balance was more favorable for the intestinal absorption of Fe, leading to increased Fe in bone. The liver results support this: Liver Fe was increased substantially by phytase supplementation at both 10 and 20 d of age (+42 % at 10 d of age) but little affected by TM supplementation. Antagonism between individual TM (for example between Zn and Fe or Mn and Fe), at sites of absorption in the intestine could also have affected the utilization of Fe in bone. Antagonism between individual TM (although not Fe) has been suggested previously (Zacharias et al., 2003; Revy et al., 2004; Jondreville et al., 2007; Bohn et al., 2008) and might arise where TM share common transporters on gut enterocytes.

There was a specific suggestion of an antagonism between Zn and Cu in the liver of phytase supplemented birds. Liver Zn at 10 d of age was unaffected by supplemental TM but was increased by phytase, whereas liver Cu was unaffected by TM supplementation but reduced (-3.5 %) by phytase. This could suggest that the increase in Zn availability effected by the phytase reduced Cu concentrations in the liver because of preferential absorbance of Zn as the most limiting TM (Bao et al., 2010). The lack of further increase in liver TM concentrations when TM were supplemented on top of phytase in CON2 may suggest that the capacity of the liver to uptake TM from the bloodstream (even for Cu) had been met by the activity of the phytase or else that an antagonism occurred that prevented further TM uptake by the liver. The study by Jondreville et al. (2007) found no effect of supplemental phytase on liver Zn or Cu concentrations but did report an association between supplemental Zn and reduced liver Cu. Other authors have also noted a negative effect of dietary Zn on liver Cu, in piglets (Zacharias et al., 2003; Revy et al, 2004) and in broilers (Ao et al., 2009) and have discussed this as a negative effect of Zn on Cu uptake by the liver. At 20 d of age, this apparent antagnosim was not evident; phytase increased liver Cu at 20 d of age. Overall, any antagonism between Zn and Cu could have been related to bird age (lower TM absorption capacity in the gut and, or alternatively, lower uptake capacity in the liver, at 10 d of age) and associated TM requirements, as well as to the deficiency level of individual TM in the basal diet. Notwithstanding these complexities, across all TM and both timepoints, the phytase increased tibia and liver Zn, Fe, and Mn, and plasma Zn. Phytase supplementation also increased feed intake which will have led to increased TM intake, but since BW and liver weight were both increased in the phytase supplemented treatments, this suggests that the increased TM concentration in the liver was mainly due to the improved availability of TM in the basal diets.

There was no consistent effect of TM source on bird responses in this study. However, imprecision about what constitutes an equivalently bioavailable dose level of organically-complexed TM vs. inorganic TM complicates the ability to draw an accurate comparison. Trace minerals from complexed sources (e.g. organic) have been shown to be more stable and bioavailable than inorganic trace minerals (sulphate or oxide sources; Bao et al., 2007; M'Sadeq et al., 2018; Aksu et al., 2012), particularly in the case of Zn (Sahin et al., 2009; Khan et al., 2024). It is also generally understood that Zn as sulfate has higher bioavailability and is absorbed to a higher degree than Zn as oxide (Veldkamp et al., 2014; Sandoval et al., 1997). For these reasons the diets containing Zn as oxide at ‘low’ and ‘high’ dose levels (treatments 4 and 5, respectively) were formulated with a higher content of added Zn than the diets containing Zn as sulfate or organic-based Zn. This was aimed at providing Zn at a more bioequivalent level across sources. This may be why no obvious effect of TM source was evident in this study.

With regard to TM dose level, there were some indications of a differential effect by dose in the TM-supplemented treatments without phytase, both at tissue (tibia Zn, Experiment 2) and bird (BW, Experiment 1) level. Previous studies have reported positive dose-response effects from supplemental Zn on growth performance and tissue concentrations of Zn, in diets without supplemental phytase (Mohanna and, Nys,1999; Sunder et al., 2008; Jondreville et al., 2007). In the phytase supplemented treatments, no effect of TM dose on bird responses was evident. This is considered to have been due to the overriding beneficial effect of the phytase.

In conclusion, when added to an all-vegetable diet containing native Zn, Mn and Cu at levels below NRC recommendations, and with varied Ca content, a novel bacterial 6-phytase variant (PhyG) improved the bioavailability of Zn, Fe, Cu and Mn from the basal ingredients and replaced the beneficial effect of supplementing these TM on growth performance during all phases. In diets already containing PhyG phytase, there was no extra benefit to growth performance and little or no extra increase in tissue TM levels from TM supplementation, regardless of TM source or dose level. Overall, the findings suggest two things: 1) That in diets without added phytase, supplementing with TM is needed for optimal growth and TM tissue utilization in young broilers up to 21 d of age but may be unnecessary during finisher phase, and; 2) In diets containing PhyG supplemented in a tiered dosing regimen by phase, the enzyme could support a reduction in commercial dose levels of added TM to all-vegetable broiler diets during all phases without loss of performance or bone mineralization. This could contribute to reducing TM excretion and improving the sustainability of broiler production.

## Declaration of competing interest

Authors declare they have no conflicts of interests.
